# Inhibition of HDAC6 Activity Protects Against Endothelial Dysfunction and Atherogenesis *in vivo*: A Role for HDAC6 Neddylation

**DOI:** 10.3389/fphys.2021.675724

**Published:** 2021-06-17

**Authors:** Yohei Nomura, Mitsunori Nakano, Hyun Woo Sung, Mingming Han, Deepesh Pandey

**Affiliations:** ^1^Department of Anesthesiology and Critical Care Medicine, Johns Hopkins University, Baltimore, MD, United States; ^2^Department of Cardiovascular Surgery, Saitama Medical Center, Jichi Medical University, Saitama, Japan; ^3^Department of Chemical and Biomolecular Engineering, Johns Hopkins University, Baltimore, MD, United States; ^4^Department of Anesthesiology, The First Affiliated Hospital of USTC, University of Science and Technology of China, Hefei, China

**Keywords:** endothelial (dys)function, vascular biology, atherosclerosis, epigentic modifier, epigenetic regulator

## Abstract

We previously reported that histone deacetylase 6 (HDAC6) has an important role in endothelial cell (EC) function *in vitro*. However, whether HDAC6 plays a role in atherogenesis *in vivo* and the mechanism(s) that control HDAC6 activity/expression in response to atherogenic stimuli are unclear. The goals of this study were to determine whether HDAC6 inhibitor tubacin attenuates atherogenesis and to elucidate specific molecular mechanism(s) that regulate endothelial HDAC6 expression/activity. We evaluated whether administration of tubacin attenuated or reversed the endothelial dysfunction and atherosclerosis induced in mice by a single intraperitoneal injection of adeno-associated viruses encoding liver-target PCSK9 gain-of-function mutant followed by a high fat diet (HFD) for 18 weeks. Tubacin significantly blunted PCSK9-induced increases in pulse wave velocity (index of vascular stiffness and overall vascular health) that are also seen in atherogenic mice. Furthermore, tubacin protected vessels from defective vasorelaxation, as evaluated by acetylcholine-mediated relaxation using wire myograph. Plaque burden defined by Oil Red O staining was also found to be significantly less in mice that received tubacin than in those that received PCSK9 alone. Inhibition of the NEDDylation pathway with MLN4924, an inhibitor of NEDD8-activating enzyme 1 (NAE1), significantly increased HDAC6 activity in HAECs. Interestingly, HDAC6 expression remained unchanged. Further, HAECs exposed to the atherogenic stimulus oxidized low-density lipoprotein (OxLDL) exhibited enhanced HDAC6 activity, which was attenuated by pretreatment with MLN4924. The HDAC6 NEDDylation molecular pathway might regulate genes related to endothelial control of vasomotor tone, reactivity, and atherosclerosis. Tubacin may represent a novel pharmacologic intervention for atherogenesis and other vasculopathies.

## Introduction

The endothelium plays a major role in the regulation of vascular homeostasis by modulating vasomotor tone, inflammation, and growth and migration of vascular smooth muscle cells. Endothelial dysfunction causes numerous abnormalities in the arterial wall (Li and Forstermann, 2009). In addition, oxidized low-density lipoprotein (OxLDL) induces pro-atherosclerotic effects in endothelial cells (ECs) by altering cell surface adhesion molecule expression (Khan et al., 1995; Liao et al., 1997), enhancing production of pro-inflammatory cytokines such as interleukin (IL)-6, TNF-α, IL-1β (Libby, 2002; Libby et al., 2002), and adhesion molecules (Khan et al., 1995); stimulating apoptosis (Dimmeler et al., 1997; Harada-Shiba et al., 1998); inducing superoxide production (Galle et al., 1995); and impairing nitric oxide (NO) production (Kugiyama et al., 1990; Simon et al., 2003). Decreases in the production and biological activity of NO lead to impaired vasodilatation and may be the earliest signs of EC dysfunction and atherogenesis (Ross, 1999).

Histone deacetylases (HDACs) have been shown to modulate atherosclerosis and EC shear stress (Choi et al., 2005; Lee et al., 2012). We have identified HDAC2 as a critical contributor to vascular homeostasis and endothelial health and shown that its inhibition leads to impaired vascular relaxation, reduced NO levels, and increased oxidative stress (Pandey et al., 2015). Conversely, increasing HDAC2 expression is endothelial-protective (Ito et al., 2002). Others have reported that SIRT1, a class III histone deacetylase, decreases acetylation of endothelial NO synthase (eNOS) post-translationally to enhance NO production (Mattagajasingh et al., 2007). Inhibition of SIRT1 increases NADPH oxidase-derived superoxide release, which leads to decreased NO bioavailability and thereby impairs aortic relaxation in rats (Mattagajasingh et al., 2007; Li et al., 2011).

HDAC6 is a structurally and functionally unique member of the HDAC family. Structurally, HDAC6 consists of two tandem deacetylase domains and a C-terminal ubiquitin binding zinc-finger domain (BUZ). Functionally, it catalyzes removal of an acetyl group from a host of cytosolic substrates other than histones, such as tubulin, HSP90, cortactin, and β-catenin (Verdel et al., 2000; Hubbert et al., 2002; Kovacs et al., 2005; Zhang et al., 2007; Li et al., 2008). Notably, unlike other HDACs, HDAC6 non-enzymatically maintains protein homeostasis by binding to ubiquitin, thereby enhancing protein clearance and degradation via the aggresome pathway (Boyault et al., 2006; Hao et al., 2013). We showed previously that increased HDAC6 activity is a major culprit in OxLDL-induced endothelial dysfunction *in vitro*. We further showed that increased activity of HDAC6 downregulates expression of endothelial cystathionine lyase γ, a major enzymatic source of the vasoactive gasotransmitter hydrogen sulfide (Kuo et al., 2016; Leucker et al., 2017). Though we have some understanding of how augmented HDAC6 activity is deleterious to cultured ECs, the role of HDAC6 in atherosclerosis *in vivo* is still unknown. In addition, it is unclear how HDAC6 activity is modulated.

Here, we show that post-translational modification of HDAC6 by small protein NEDD8 regulates its activity. Although the process of conjugation is analogous to ubiquitination, NEDDylation regulates broad range of its substrates functions such as activity, abundance, stability, and subcellular localization (Hershko and Ciechanover, 1998; Hershko, 2005; Oved et al., 2006; Enchev et al., 2015). Indeed, inhibition of NEDDylation attenuates NFκB-mediated release of proinflammatory cytokines in macrophages and human ECs (Chang et al., 2012; Ehrentraut et al., 2013). As we previously reported, inhibition of NEDDylation by MLN4924 augments HDAC2 abundance *in vitro* and *in vivo* and protects impaired endothelium-dependent vascular relaxation induced by OxLDL (Pandey et al., 2015). More importantly, a recent study has described a protective role of the DeNeddylase enzyme COPS9 in atherogenesis (Asare et al., 2017). Small molecule inhibitors of NEDDylation activating enzyme 1 (NAE1) may therefore represent a novel therapy for endothelial dysfunction and atherosclerosis.

In this study, we extend our previous findings of HDAC6 to establish its contribution to the endothelial dysfunction that underlies development of atherosclerosis *in vivo*. Notably, we investigated the use of tubacin, a small molecule inhibitor of HDAC6, in a mouse model of atherosclerosis. We then evaluated aortic stiffness and vascular relaxation—both important metrics of endothelial dysfunction—and measured atherosclerotic plaque load. Further, we identified the post-translational mechanism that regulates HDAC6 activity in human aortic endothelial cells (HAECs). Our results could shed light on the previously observed anti-atherogenic effects of MLN4924.

## Materials and Methods

### Reagents

All experimental procedures involving mice were approved by the Institutional Animal Care and Use Committee (IACUC) at The Johns Hopkins University School of Medicine. Eight to ten weeks old C57BL/6 mice were purchased from Jackson Laboratory (Bar Harbor, ME). Unless otherwise stated, all reagents were obtained from Sigma (St. Louis, MO). Antibodies to hemagglutinin (HA; C29F4), HDAC6, NEDD8, p-eNOS (T495) and FLAG were purchased from Cell Signaling (Danvers, MA). NOS3 (A-9) antibody was purchased from Santa Cruz Biotechnology (Dallas, TX). Acetylated α-tubulin antibody was purchased from Abcam (ab24610; Cambridge, MA), total α-tubulin antibody was purchased from Invitrogen (62204; Waltham, MA), and Lipofectamine 2000 was purchased from Life Technologies (Waltham, MA). Tubacin was purchased from Enzo Life Sciences (Farmingdale, NY). Fresh batches of oxidized low-density lipoprotein (OxLDL) was purchased from Alfa Aesar (a Johnson Matthew company).

### Cell Culture

#### Endothelial Cell Lines

HAECs were purchased from PromoCell and maintained in Endothelial Cell Growth Medium MV 2 (PromoCell) according to the supplier’s protocol at 37°C in a humidified, 5% CO_2_ atmosphere. Cells were passaged at 90% confluency. MLN4924 were purchased from Enzolifesciences and reconstituted in DMSO. HAECs were incubated in EC growth media with either DMSO (control) or MLN4924 (0.1–1 μM) for 24 h.

#### Immortal Cell Lines

Human embryonic kidney cells (HEK293A) were purchased from Life Technologies and maintained in DMEM (Gibco) supplemented with 10% fetal bovine serum (Gibco) and 1% penicillin-streptomycin (Corning). Cells were passaged at 75% confluency.

### DNA Constructs

Full-length FLAG epitope-tagged HDAC6 was a kind gift from Eric Verdin (Addgene plasmid #13823). The catalytically inactive mutant of HDAC6 (H216/611A, Addgene plasmid #30483) and C-terminal zinc finger domain (binder of ubiquitin zinc finger, BUZ Addgene plasmid #30484) deleted mutant were kind gifts from Tso-Pang Yao (Kawaguchi et al., 2003). All other constructs were cloned in-house by using topo-directional cloning (Invitrogen).

### Atherogenesis Model

Eight weeks old C57BL/6 wild-type (WT) male mice were administered a single tail-vein injection of 1 × 10^11^ vector genomes of adeno-associated virus (AAV) encoding a gain-of-function PCSK9 variant (D377Y). Then they were randomly assigned to receive vehicle or tubacin (intraperitoneal, 0.5 mg/kg daily) and fed a high-fat diet (HFD) for 18 weeks (Goettsch et al., 2016). A separate set of control mice did not receive the AAV-PCSK9 injection. Pulse wave velocity (PWV) and body weight were measured every 2 weeks in all mice. We have described this model in detail in our previous study (Hori et al., 2020).

### Non-invasive Pulse Wave Velocity (PWV) Measurements

A high-frequency, high-resolution Doppler spectrum analyzer (DSPW, Indus Instruments, Houston, TX, United States) was used. Mice were anesthetized with 1.5% isoflurane and placed in a supine position on a heated pad equipped with echocardiogram (EKG) capability. The animals were allowed to stabilize to a physiologic heart rate before a 20 MHz probe was used to measure the descending aortic and abdominal aortic flow velocities. The time from the R wave of the EKG to the start of pulse waveform for each measurement location was calculated by using a real-time signal acquisition and spectrum analyzer system.

### Pathological Assessment of Plaque in Aortas

Aorta, from aortic root to iliac artery bifurcation, was carefully dissected, perfused with Krebs solution, and fixed with 4% paraformaldehyde overnight. The aorta was opened longitudinally and pinned onto a wax surface by microneedles. Images of the submerged vessels were captured with a digital camera. The lipid-rich intraluminal lesions were stained with Oil red O (Sigma). Digitized images were transferred to a computer and analyzed with NIH Java Image (ImageJ, version 1.42n). The amount of aortic atheroma in each animal was measured as percent lesion area per total area of the aorta. Aortic root was embedded in OCT compound (Fisher Scientific) and cross-section was fixed with 10% formalin and stained with Oil red O.

### Oil Red O Staining

Oil red O was purchased from Sigma-Aldrich (catalog no. MAK194) and the staining was performed according to manufacturer’s instruction. Briefly, gross aortas or the aortic sections were fixed with 10% formalin for 30 min. After washing formalin with water twice, the samples were incubated in 60% isopropanol for 5 min. Isopropanol was discarded and the samples were further incubated in oil red o stain solution (prepared in 100% isopropanol) for 20 min. The samples were washed for 5 times with water. The gross aortas were pinned to the wax surface and the cross section of aortic root was mounted with a mounting media (FluorSave) in a coverslip and images were taken under magnifying scope using camera and phase microscope, respectively. Aortic roots were frozen using the Tissue-Tek optimum cutting temperature (O.C.T) formulation purchased from VWR, sectioned, stained with Oil Red O and image were taken using phase microscope.

### Force Tension Myography

Mouse aorta was isolated and cleaned in ice-cold Krebs-Ringer-bicarbonate solution containing the following (in mM): 118.3 NaCl, 4.7 KCl, 1.6 CaCl_2_, 1.2 KH_2_PO_4_, 25 NaHCO_3_, 1.2 MgSO_4_, and 11.1 dextrose. The aorta was immersed in a bath filled with constantly oxygenated Krebs buffer at 37°C. Equal-sized thoracic aortic rings (2 mm) were mounted under microscopy to ensure no damage to the smooth muscle or endothelium. One end of each aortic ring was connected to a transducer, and the other to a micromanipulator. The aortas were passively stretched to an optimal resting tension with the micromanipulator. Aorta was passively stretched to an optimal resting tension using the micromanipulator, after which a dose of 60 mM KCl was administered and repeated after a wash with Krebs buffer. After these washes, all vessels were allowed to equilibrate for 20–30 min in the presence of indomethacin (3 μM). Phenylephrine (1 μM) was administered to induce vasoconstriction. A dose- dependent response (1 nM–10 μM), with the muscarinic agonist, ACH or nitric oxide donor, SNP, was then performed as necessary. Relaxation responses were calculated as a percentage of tension following pre-constriction. Sigmoidal dose-response curves were fitted to data with the minimum constrained to 0. Two to four rings were isolated from each animal and the number of animals in each group (n) was 6.

### Immunoprecipitation and Western Blotting

After 48 h of HEK293A cell transfection, cells were lysed in a modified ice-cold RIPA lysis buffer consisting of 20 mM Tris–HCl at pH 7.5, 150 mM NaCl, 1 mM EDTA, 1 mM EGTA, 0.3% NP40, 1% sodium deoxycholate, 1 mM Na_3_VO_4_, 2.5 mM sodium pyrophosphate, 1 mM β-glycerophosphate, 1 μg/mL leupeptin, and a 1:1,000 diluted protease inhibitor cocktail (Sigma). For immunoprecipitation studies, whole cell lysates were centrifuged at 14,000 × *g* and supernatants were precleared by incubation with Protein A/G-agarose beads for 2 h at 4°C with rocking. Agarose beads were then pelleted by centrifugation at 1,000 × *g*. HA-NEDD8 in precleared lysates was immunoprecipitated by incubation overnight at 4°C with rocking after addition of anti-HA antibody (1:150). Immune complexes were eluted in sodium dodecyl sulfate (SDS) sample buffer, boiled for 5 min, and then loaded onto polyacrylamide gels for SDS–PAGE. Western blot analysis was performed by transferring proteins from the SDS gel onto a nitrocellulose membrane. Protein bands were visualized by secondary antibodies conjugated to alkaline phosphatases.

### Statistical Analysis

All statistical analyses were carried out in Prism 7 for Mac (GraphPad Software Inc., San Diego, CA) and Excel version 14.1.3 (Microsoft, Redmond, WA) statistical analysis software. The results are expressed as mean ± standard error of the mean (mean ± SEM). Comparisons of all experimental data sets, groups, and pairs of data sets were analyzed with one-way ANOVA followed by the Bonferroni *post-hoc* test for multiple comparisons. A value of *p* < 0.05 was considered statistically significant. Myograph data were analyzed in STATA VERSION 15 software (STATA Corporation, College Station, TX) by using a general linear model with group as a factor and dose as a repeated measure. We applied *post-hoc* tests for each dose using linear combination based on this model.

## Results

### Pharmacologic Inhibition of HDAC6 With Tubacin Attenuates Vascular Stiffness, Improves Relaxation, and Decreases Plaque Lesions in Atherosclerotic Mice

**FIGURE 1:**
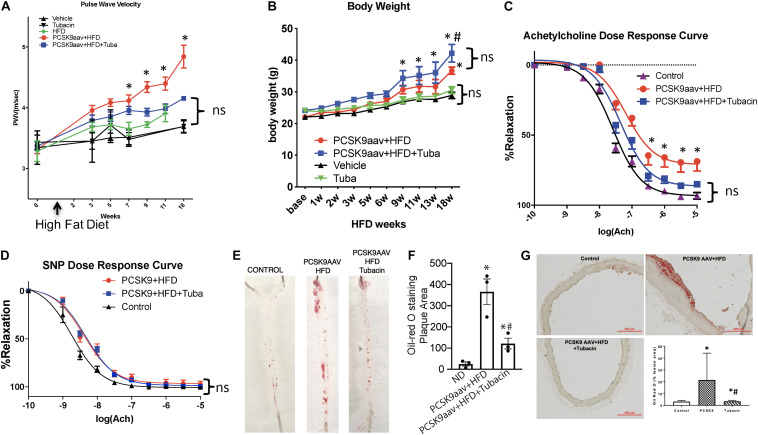
Pharmacologic inhibition of HDAC6 attenuates elevated vascular stiffness and protects impaired endothelial-dependent vascular relaxation in atherogenic mice. Six- to eight-week-old C57BL/6 mice were injected with adeno-associated virus (AAV) encoding a PCSK9 gain-of-function mutant and then were fed a high-fat diet (HFD) for 18 weeks in the presence or absence of the HDAC6 inhibitor tubacin (0.5 mg/kg daily). **(A)** Pulse wave velocity (PWV) and **(B)** body weight was measured every 2 weeks until the end of the diet regimen (18 weeks). ^∗^*p* < 0.05 vs. vehicle group; *n* = 10–12. Vascular reactivity in response to **(C)** acetylcholine and **(D)** sodium nitroprusside (SNP) was measured by wire myography of isolated aortic rings. ^∗^*p* < 0.05 vs. control group; *n* = 6–8 mice. **(E)** Oil Red O staining was used to determine plaque burden in isolated aortas. **(F)** Quantification of plaque area presented in **(E)**. ^∗∗^*p* < 0.05 vs. normal diet group; ^#^*p* < 0.05 vs. PCSK9aav + HFD group; *n* = 3. **(G)** Frozen aortic root cross sections were stained with Oil Red O. The graph in the lower right quadrant shows quantification of plaque area. ^∗^*p* < 0.05 vs. normal diet group; ^#^*p* < 0.05 vs. PCSK9aav + HFD group; *n* = 3.

To determine whether HDAC6 inhibition improves vascular function, we examined the effect of HDAC6 inhibitor tubacin on vascular stiffness in atherogenic mice. Aortic PWV, a reciprocal index for vascular stiffness and overall vascular health, rose significantly in both vehicle- and tubacin-treated mice after PCSK9 transduction as compared to that in control (uninduced) mice with or without HFD (Figure 1A). However, PWV was significantly attenuated in the tubacin-treated group as compared to that in the vehicle-treated group (Figure 1A). Body weight significantly increased in PCSK9 injected and HFD fed mice as expected and interestingly these mice treated with tubacin weighed slightly but significantly more (Figure 1B). Further, as shown in Figure 1C, aortic rings from atherogenic C57BL/6 mice exhibited impaired acetylcholine-induced (endothelial-dependent) vasorelaxation after the HFD (Log EC_50_: -7.19 ± 0.11, 95%CI -7.40 to -6.97) compared to that of uninduced controls (Log EC_50_: -7.58 ± 0.04, 95%CI -7.67 to -7.50). However, tubacin rescued vasorelaxation (Log EC_50_: -7.35 ± 0.06, 95%CI -7.47 to -7.22, *p* < 0.001). Sodium nitroprusside-induced (endothelial-independent) relaxation was not significantly different (Figure 1D). Oil Red O staining revealed a striking increase in plaque burden in WT mice subjected to the atherogenesis model and markedly lower plaque formation in tubacin-treated mice (Figures 1E–G). Quantification of cross-sections of Oil-Red O–stained aortic root showed significantly elevated plaque burden in atherogenic mice that was attenuated with daily tubacin toward the end of the HFD regimen. To determine whether atheroprotective effects of HDAC6 inhibition involved attenuation of inflammation, we measured whether intracellular adhesion molecules 1 (ICAM1) is attenuated in aortas isolated from control and tubacin treated atherogenic mice. As shown in Supplementary Figure 1, our data show decreased trend in ICAM1 expression in tubacin treated group. However, the differences was not statistically significant.

### NEDDylation Pathway Inhibition Attenuates HDAC6 Activity

**FIGURE 2:**
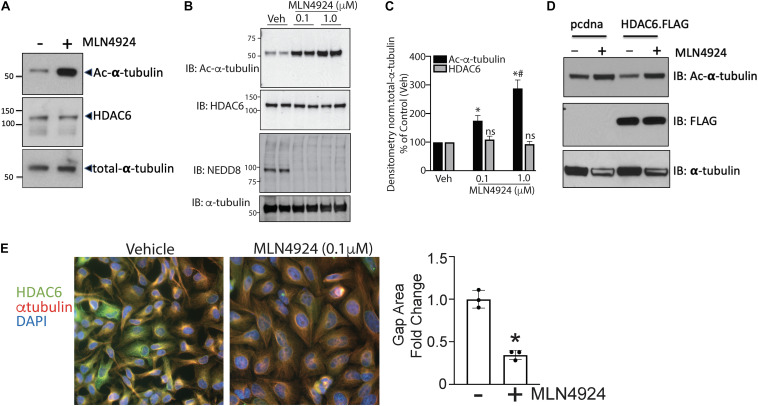
Pharmacologic inhibition of NEDDylation pathway regulates endothelial HDAC6 activity and modulates α-tubulin organization. **(A)** Human aortic endothelial cells (HAECs) were exposed to either DMSO or MLN4924 (1 μM) for 18 h, and cell lysates were subjected to western blotting with antibodies to acetylated α-tubulin, HDAC6, or total α-tubulin. **(B)** Similar experiments were performed with two doses of MLN4924 (0.1 and 1 μM); *n* = 3. **(C)** Densitometric analysis of HDAC6 and acetylated α-tubulin levels from panels **(A,B)**. ^∗^*p* < 0.05 vs. vehicle group; ^#^*p* < 0.05 vs. MLN4925 (0.1 μM) group; *n* = 3. **(D)** HEK293 cells were transfected with empty vector (pcdna3.1) or FLAG-tagged HDAC6. After 24 h, MLN4924 or DMSO (control) was added to the media and cultured for another 18 h. Cell lysates were immunoblotted (IB) with acetylated α-tubulin and FLAG antibodies. **(E)** Left: A confluent monolayer of HAECs was exposed to DMSO or MLN4924 (1 μM) for 18 h. Cells were immunofluorescently stained for HDAC6 (green), α-tubulin (red), and DAPI (blue). Right: Quantification of gap area between adjacent cells normalized to the number of nuclei. ^∗^*p* < 0.05 vs. control group; *n* = 3.

Previous studies have shown that MLN4924, an inhibitor that targets NAE1, provides protection against endothelial dysfunction in response to oxidative injury, halts early atherosclerosis, and inhibits inflammation in macrophages and ECs. Further, elevated HDAC6 activity has been implicated in the development of atherosclerosis, and tubacin enhances the atheroprotective effects of eNOS. Therefore, we next tested whether crosstalk occurs between the NEDDylation pathway and HDAC6. We first evaluated the effects of MLN4924 on HDAC6 activity and expression in HAECs. Because HDAC6 is a microtubule-associated deacetylase, we assessed its activity by measuring levels of acetylated α-tubulin (Hubbert et al., 2002). Interestingly, acetylated α-tubulin levels increased significantly while HDAC6 expression remained unchanged in MLN4924-exposed HAECs (Figure 2A). Further, MLN4924 dose-dependently increased acetylated α-tubulin (Figure 2B). The effectiveness of NEDDylation pathway inhibitor MLN4924 was demonstrated by immunoblotting for NEDD8. We found a marked decrease in NEDDylated substrates, suggesting that MLN4924 reduces NEDD8 conjugation (Figures 2B,C). Further, MLN4924 completely blocked the ectopically transfected HDAC6 (FLAG epitope-tagged) activity in HEK293 cells, as indicated by reciprocal increases in levels of acetylated tubulin (Figure 2D). A major component of microtubules, α-tubulin forms the cellular cytoskeletal system and plays critical role in endothelial migration, cell shape, and adhesion, all critical in the development of atherosclerotic plaques (Hubbert et al., 2002; Hashimoto-Komatsu et al., 2011). Here, immunofluorescence with α-tubulin antibody revealed quiescent ECs with more concentrated radiating α-tubulin and smaller nuclei than those of MLN4924-treated ECs, which showed reorganization of spread-out α-tubulin and enlarged round nuclei (Figure 2E). We also observed significantly fewer gaps between adjacent cells in MLN4924-treated ECs (Figure 2E).

**FIGURE 3:**
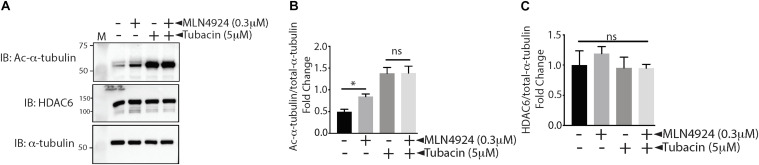
MLN4924 fails to increase acetylated tubulin in tubacin-pretreated HAECs. **(A)** HAECs were pretreated with tubacin for 4 h before addition of MLN4924 (1 μM). After an 18 h incubation, cells were immunoblotted (IB) with antibodies to acetylated α-tubulin, HDAC6, and total α-tubulin. **(B,C)** Densitometric analysis of HDAC6 and acetylated α-tubulin levels presented in **(A)**. ^∗^*p* < 0.05 vs. DMSO (control) group; *n* = 3.

In our study, we used the acetylation status of α-tubulin, a well-known HDAC6 substrate, to measure HDAC6 activity indirectly. To determine whether the increase in acetylation of α-tubulin that occurred when ECs were treated with MLN4924 was indeed due to decreased HDAC6 activity, we measured the effect of MLN4924 in tubacin-pretreated (HDAC6-inhibited) HAECs. As shown in Figures 3A,B, MLN4924 failed to increase acetylated α-tubulin levels in tubacin-pretreated HAECs, which showed a robust increase in acetylated α-tubulin levels. HDAC6 levels remained unchanged with MLN4924, regardless of whether cells were pretreated with tubacin (Figure 3C). These data further confirm that MLN4924 decreases HDAC6 activity.

### HDAC6 Is a NEDD8 Substrate and the Potential NEDD8 Site Is Located Within HDAC6 the BUZ Domain

**FIGURE 4:**
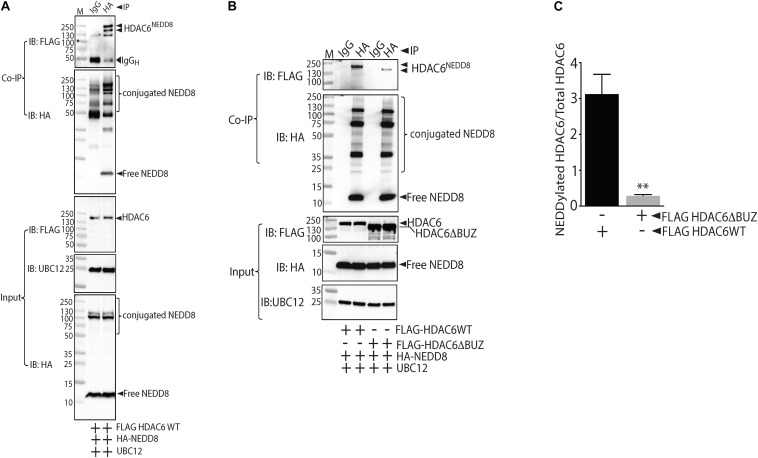
NEDD8 binds HDAC6 within the ubiquitin binding BUZ domain. **(A)** HEK293 cells expressing FLAG-tagged full-length HDAC6 (FLAGHDAC6WT), HA-NEDD8, and UBC12 were immunoprecipitated (IP) with either isotype control IgG or HA antibodies. The purified conjugates were immunoblotted (IB) with FLAG, HA, or UBC12 antibodies. **(B)** HEK293 cells expressing FLAG-tagged full-length HDAC6 (FLAGHDAC6WT) or ubiquitin binding domain truncated HDAC6 mutant (FLAGHDAC6ΔBUZ) together with HA-NEDD8 and UBC12 were immunoprecipitated with either isotype control IgG or HA antibodies. The purified conjugates were immunoblotted with FLAG, HA, or UBC12 antibodies. **(C)** Densitometric analysis of NEDDylated FLAGHDAC6 WT and FLAGHDAC6ΔBUZ normalized to total HDAC6. ^∗∗^*p* < 0.05 vs. FLAGHDAC6 WT group; *n* = 3.

Our data showed that NEDDyation pathway inhibitor MLN4924 inhibited HDAC6 activity. Next we needed to ascertain whether MLN4924 inhibited direct NEDDylation of HDAC6 or NEDDylation of an HDAC6 regulator. Therefore, we first determined whether HDAC6 is a direct substrate of NEDD8. Indeed, co-immunoprecipitation experiments showed that HDAC6 forms stable complexes with NEDD8 in HEK293 cells that co-express FLAG-tagged HDAC6 and HA-tagged NEDD8 (Figure 4A). UBC12, a NEDD8 E2 conjugating enzyme was transfected in all groups to enhance conjugation of NEDD8 to its substrates. We then sought to identify the specific motif within HDAC6 responsible for NEDD8 conjugation. The NEDD8 conjugation site prediction software NEDDYPREDDY predicted clusters of lysine as a potential NEDDylation site in HDAC6. Therefore, we used full-length HDAC6 (FLAGHDAC6-FL) and a C-terminal BUZ truncation mutant of HDAC6 (FLAGHDAC6 ΔBUZ) in co-immunoprecipitation experiments with HA-NEDD8. NEDD8 conjugation was nearly abolished in the HDAC6 ΔBUZ mutant suggesting that the NEDDylation site in HDAC6 is located in the ubiquitin-binding domain of HDAC6 (Figures 4B,C).

### Atherogenic- and OxLDL-Increased HDAC6 Activity in ECs Is Abrogated by NAE1 Inhibitor MLN4924

**FIGURE 5:**
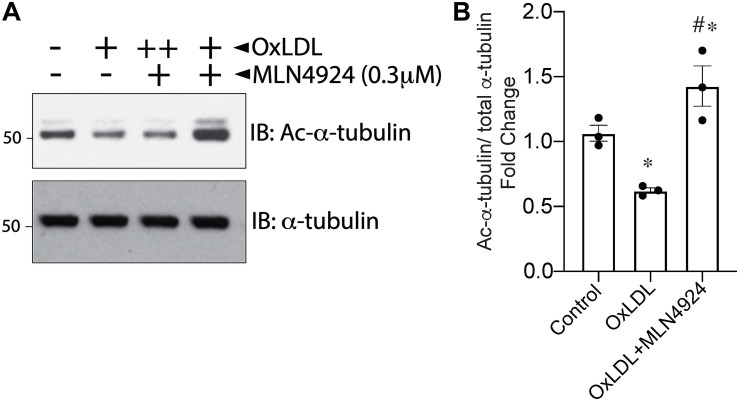
Pharmacologic inhibition of NEDDylation pathway blocks OxLDL-induced HDAC6 activity. **(A)** HAECs pretreated with MLN4924 (1 μM) for 2 h were exposed to either phosphate-buffered saline (control) or increasing concentrations of OxLDL (50, 100 μg/mL) for 6 h. Cell lysates were immunoblotted (IB) with acetylated α-tubulin and total α-tubulin antibodies. **(B)** Densitometric analysis of acetylated α-tubulin. ^∗^*p* < 0.05 vs. control group; ^#^*p* < 0.05 vs. OxLDL group; *n* = 3.

Our previous studies showed that the atherogenic stimulus OxLDL triggers global protein NEDDylation and enhances HDAC6 deacetylation activity in HAECs (Pandey et al., 2015; Leucker et al., 2017). However, whether NEDDylation was the link between increased HDAC6 with OxLDL was unknown. Therefore, we examined whether NEDDylation plays any role in OxLDL-mediated activation of HDAC6. Indeed, MLN4924 not only blocked the stimulatory effect of OxLDL on HDAC6 activity in HAECs (as evidenced by an increase in acetylated α-tubulin) but further reduced it below control levels (Figures 5A,B). These findings further suggest that NEDDylation of HDAC6 may be responsible for impairment of the vascular response by oxidative injury.

## Discussion

In this study we found that pharmacologic inhibition of HDAC6 with tubacin restores endothelial-dependent relaxation, prevents the development of vascular stiffness—one of the integrated measures of vascular health *in vivo*—and significantly reduces plaque burden in a mouse model of atherosclerosis. We also define here a novel post-translational mechanism that can control HDAC6 activity in the context of oxidative injury whereby NEDD8 is conjugated to lysine located within the C-terminal ubiquitin binding domain of HDAC6.

In our previous study we showed that the atherogenic stimulus OxLDL triggers endothelial dysfunction by downregulating expression of atheroprotective enzyme endothelial cystathionine lyase γ, which produces vasoactive hydrogen sulfide through activation of its transcriptional regulator HDAC6. Those results implicated post-translational modification via NEDDylation (covalent attachment of NEDD8 protein) and subsequent degradation of HDAC2 as causative factors (Pandey et al., 2015; Leucker et al., 2017). In contrast to its effect on HDAC2 levels, NEDDylation of HDAC6 did not alter HDAC6 levels but rather increased its activity. These effects further highlight the diverse cellular effects of NEDDylation. Our findings are consistent with a non-biased targeted proteomic study that identified HDAC2 and HDAC6 as the only two potential HDAC substrates of NEDD8 (Jones et al., 2008). One of the limitations of our current study is that we have not yet identified the specific lysine residue(s) in HDAC6 that covalently bind to NEDD8. It would be interesting to know whether mutation of the NEDD8 conjugation site in mice and in cultured HAECs using CRISPR/Cas9 would provide protection against endothelial dysfunction and atherogenesis. Using NEDDylation site predictor software and domain truncation, we have identified the C-terminal ubiquitin binding domain of HDAC6 as a potential site for NEDD8 conjugation. Future studies are warranted to understand the mechanism(s) by which NEDDylation affects HDAC6 activity in ECs.

A recent study identified tubacin as a potent inducer of eNOS expression and NO production in EC lines *in vitro*, and in mice *in vivo* (Chen et al., 2019). Interestingly, this effect was independent of tubacin’s ability to inhibit HDAC6. That study exposes a limitation of our own, in that the protective effect of tubacin against endothelial dysfunction and atherogenesis could be independent of HDAC6 inhibition. Future studies with HDAC6 knockout mice are needed to clarify this important issue. Nonetheless, these studies, which show that tubacin increases athero-protective NO and hydrogen sulfide production in ECs, and blocks atherosclerosis (current study), further strengthen the case for tubacin as a therapeutic application to prevent/reverse atherogenesis.

MLN4924, which inhibits the NEDDylation pathway, has been shown to attenuate inflammation and prevent atherosclerosis (Ehrentraut et al., 2013; Asare et al., 2017). In current study eNOS expression and phosphorylation was unaffected in HAEC treated with MLN4924 (Supplementary Figure 2). Studies are warranted to determine the specific effects of HDAC6 inhibition by MLN4924 on endothelial function and atherosclerosis. The identification of NEDDylation site and generation of mutant mice will facilitate to address this important question.

Endothelial barrier integrity is compromised in the process of atherosclerosis, and selective inhibition of HDAC6 in human pulmonary artery ECs has been shown to prevent TNF-α–induced EC barrier dysfunction and endotoxin-induced pulmonary edema (Yu et al., 2016). ECs are known to respond to altered fluid shear stress by altering their cellular and nuclear shape to align with the direction of flow. Microtubules are critical for this process. One of the key functions of the EC morphologic change is the transient increase in intracellular calcium that activates eNOS to release NO. In this study, we observed striking changes in EC morphology, tubulin reorganization, and nuclear shape with MLN4924 treatment. Although we did not measure the functional consequence of these changes, the shortened gaps between ECs suggest that NEDDylation could have important role in maintaining the barrier integrity of ECs. This finding is promising, given the clinical importance of endothelial barrier integrity in the regulation of inflammation and atherosclerosis. Indeed, inhibition of the deNEDDylation enzyme CSN5 has been implicated in loss of EC barrier function (Majolée et al., 2019). Further, MLN4924 increases the expression of β-catenin, which is a common substrate of cullin-ring ligases and HDAC6, and interacts with VE-cadherin at EC junctions to maintain barrier integrity, suggesting a link between NEDDylation, cell-to-cell adhesion, and barrier function (Gu et al., 2014; Tran et al., 2016).

In current study tubacin treatment significantly reduced aortic stiffness, a metric of global integrated vascular health *in vivo*, in mice that were induced to develop atherosclerosis. Our previous study shows that the elevated aortic stiffness in atherogenic mice was independent of changes in mean arterial blood pressure (Hori et al., 2020). The attenuation of aortic stiffness in tubacin treated atherogenic mice found in current study is therefore unlikely to be mediated by changes in blood pressure. The mechanism by which HDAC6 inhibition exerts protective effect on endothelial function has been shown to involve enhanced production of vasodilator gas hydrogen sulfide by cystathionine gamma lyase (Leucker et al., 2017). However, whether this holds true in atherogenesis is yet to be determined.

**FIGURE 6:**
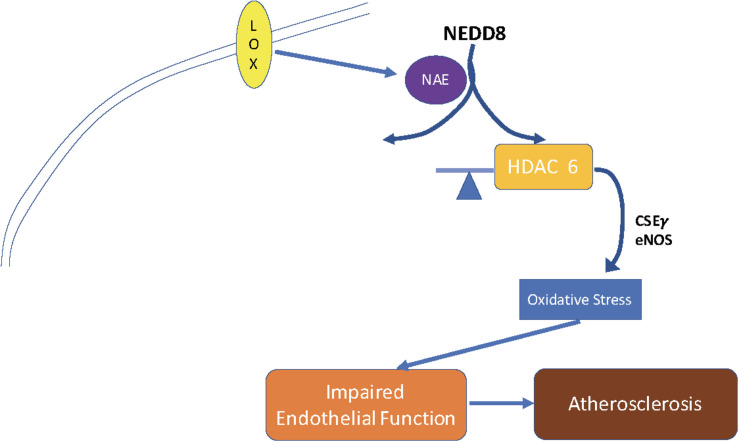
Schematic of proposed mechanism for the role of HDAC6 in endothelial dysfunction and atherogenesis. OxLDL stimulates HDAC6 activity by enhancing HDAC6 NEDDylation. This leads to suppression of the endothelial enzymes NOS and CSEγ, thereby reducing NO and H_2_S. These events result in EC dysfunction and atherosclerosis.

Taken together, our findings suggest a novel molecular pathway that influences endothelial tone, reactivity, and atherogenesis Figure 6. These results provide insight that could help us to elucidate the mechanism by which genes control atherogenesis and vasculopathies. Further, blocking HDAC6 activity directly with tubacin or indirectly by blocking its post-translational modifier NEDD8 with MLN4924, provides options for new therapeutic strategies to treat or reverse endothelial dysfunction and atherosclerosis.

## Data Availability Statement

The raw data supporting the conclusions of this article will be made available by the authors, without undue reservation.

## Ethics Statement

The animal study was reviewed and approved by the Johns Hopkins University Animal Care and Use Committee (ACUC).

## Author Contributions

DP: conception and design and drafting the manuscript for important intellectual content. YN, MN, HSW, MH, and DP: analysis and interpretation. All authors have approved it for publication.

## Conflict of Interest

The authors declare that the research was conducted in the absence of any commercial or financial relationships that could be construed as a potential conflict of interest. The handling editor declared a shared affiliation with one of the author DP.
