# Supplementary material for: Inhibition of HDAC6 Activity Protects Against Endothelial Dysfunction and Atherogenesis in vivo: A Role for HDAC6 Neddylation
Source: Front Physiol. 2021 Jun 17;12:675724. doi: 10.3389/fphys.2021.675724 (PMC8245780; doi:10.3389/fphys.2021.675724)
Supplement: Supplementary file 1 [file Data_Sheet_1.pdf]

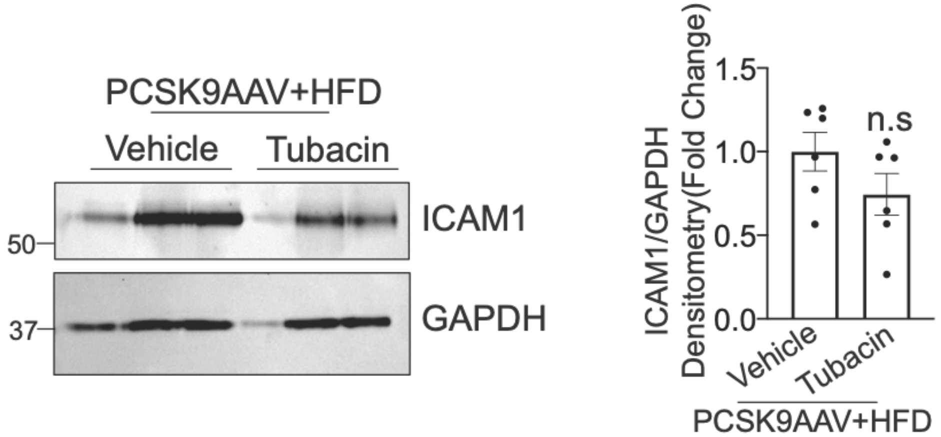

Supplemental Figure I. Effect of pharmacologic inhibition of HDAC6 on aortic inflammatory marker ICAM1 in atherogenic mice. Six- to eight-week-old C57BL/6 mice were injected with adeno-associated virus (AAV) encoding a PCSK9 gain-of-function mutant and then were fed a high-fat diet (HFD) for 18 weeks in the presence or absence of the HDAC6 inhibitor tubacin (0.5 mg/kg daily). Isolated aortas were subjected to immunoblotting using anti-ICAM1 and anti-GAPDH antibodies. Quantification of western blots are presented on right (n.s=not significant).

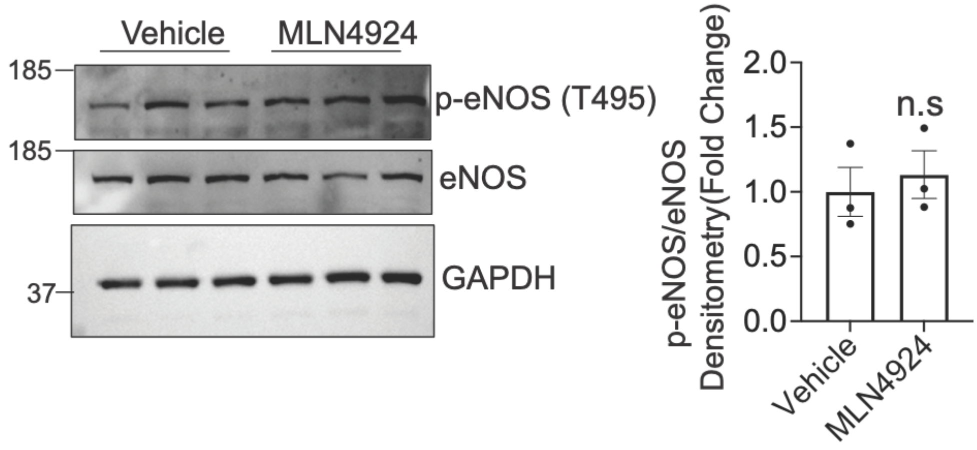

Supplemental Figure II. Effect of MLN4924 on eNOS phosphorylation in quiescent endothelial cells. Human aortic endothelial cells (HAECs) were exposed to either DMSO or MLN4924 (1 μM) for 18 h, and cell lysates were subjected to western blotting with antibodies to phosphor-eNOS (T495), eNOS, or GAPDH. Quantification of western blots are presented on right (n.s=not significant).
